# A *De Novo* Noncoding *RARB* Variant Associated with Complex Microphthalmia Alters a Putative Regulatory Element

**DOI:** 10.1155/2024/6619280

**Published:** 2024-01-27

**Authors:** Maria R. Replogle, Samuel Thompson, Linda M. Reis, Elena V. Semina

**Affiliations:** 1 Department of Ophthalmology and Visual Sciences, Medical College of Wisconsin, Milwaukee, WI, USA; 2 Department of Pediatrics and Children’s Research Institute, Medical College of Wisconsin and Children’s Hospital of Wisconsin, Milwaukee, WI, USA

## Abstract

Retinoic acid receptor beta (*RARB*) is a transcriptional regulator crucial for coordinating retinoic acid- (RA-) mediated morphogenic movements, cell growth, and differentiation during eye development. Loss- or gain-of-function *RARB* coding variants have been associated with microphthalmia, coloboma, and anterior segment defects. We identified a *de novo* variant c.157+1895G>A located within a conserved region (CR1) in the first intron of *RARB* in an individual with complex microphthalmia and significant global developmental delay. Based on the phenotypic overlap, we further investigated the possible effects of the variant on mRNA splicing and/or transcriptional regulation through *in silico* and functional studies. *In silico* analysis identified the possibility of alternative splicing, suggested by one out of three (HSF, SpliceAI, and MaxEntScan) splicing prediction programs, and a strong indication of regulatory function based on publicly available DNase hypersensitivity, histone modification, chromatin folding, and ChIP-seq data sets. Consistent with the predictions of SpliceAI and MaxEntScan, *in vitro* minigene assays showed no effect on *RARB* mRNA splicing. Evaluation of CR1 for a regulatory role using luciferase reporter assays in human lens epithelial cells demonstrated a significant increase in the activity of the *RARB* promoter in the presence of wild-type CR1. This activity was further significantly increased in the presence of CR1 carrying the c.157+1895G>A variant, suggesting that the variant may promote *RARB* overexpression in human cells. Induction of *RARB* overexpression in human lens epithelial cells resulted in increased cell proliferation and elevated expression of *FOXC1*, a known downstream target of RA signaling and a transcription factor whose down- and upregulation is associated with ocular phenotypes overlapping the *RARB* spectrum. These results support a regulatory role for the CR1 element and suggest that the *de novo* c.157+1895G>A variant affecting this region may alter the proper regulation of *RARB* and, as a result, its downstream genes, possibly leading to abnormal development.

## 1. Introduction

Defects in retinoic acid (RA) signaling during vertebrate eye development contribute to a broad range of developmental ocular disorders, including microphthalmia, anophthalmia, and coloboma (MAC) spectrum, anterior segment dysgenesis (ASD), and congenital glaucoma, and are often associated with nonocular malformations affecting the brain, heart, diaphragm, lungs, and limbs [1, 2]. Retinoic acid receptors function as transcriptional regulators within the RA signaling pathway, which is essential for coordinating the proper formation of structures derived from the neuroectoderm, surface ectoderm, and periocular mesenchyme (POM) during vertebrate eye development [3, 4]. RA serves as a ligand for two families of nuclear transcriptional regulators: retinoic acid receptors (RARA, RARB, and RARG), which bind all-trans or 9-*cis*-RA, and retinoid X receptors (RXRA, RXRB, and RXRG), which bind 9-*cis*-RA only [5]. RAR and RXR subtypes differentially heterodimerize to form ligand-bound complexes which bind specific consensus regulatory elements to selectively control the expression of RA-target genes associated with organogenesis, morphogenesis, cell growth, differentiation, and homeostasis [1, 6]. Cell type-specific expression of RAR and RXR subtypes, as well as the balance of RA synthesis and degradation, establishes spatiotemporal signaling gradients crucial for fine-tuning the functional output of RA-mediated gene networks during development.

All RAR and RXR subtypes are expressed in the eye, albeit with dynamic cell type- and tissue-specific distribution patterns over the course of ocular development [7, 8]. Specifically, *RARB* is expressed in the lens, choroid, retinal pigment epithelium, and inner neuroblastic layer of the retina, with the most predominant expression reported in the POM and its derivatives, including sclera, iris, and ciliary body [8, 9]. Genetic ablation of *Rarb* and *Rarg* in murine POM cells resulted in significant anterior segment defects, including agenesis of the iris, a smaller conjunctival sac, and thickening of the mesenchyme component of the cornea, as well as persistence and hyperplasia of the vitreous body, shortening of the ventral retina and ventral rotation of the eye [10]. Similar ocular phenotypes were also reported in *Rarb*-null and *Rarb*/*Rarg*-null mice [7, 11, 12].

While recessive truncating variants in *RARB* were identified first in a family with four siblings affected with PDAC syndrome (pulmonary hypoplasia or agenesis, diaphragmatic hernia or eventration, A/M, and cardiac defects), all subsequent variants have exhibited a dominant mechanism with more variable nonocular anomalies termed Microphthalmia, syndromic 12 (MCOPS12; MIM 615524) [13, 14]. To date, 28 unique dominant variants have been reported, the majority of which are missense alleles within the ligand binding domain. The Arg387 residue represents a hotspot, with three different recurrent missense alleles (p.Arg387Cys, p.Arg387Ser, and p.Arg387Leu) reported in over 18 families [13, 14]. For dominant alleles, gain-of-function is the most common mechanism, followed by dominant-negative effects [14]. Developmental eye anomalies are the most penetrant features of MCOPS12, reported in 87% of individuals. While severe gross motor (nonambulatory) and language (nonverbal to single words) delays were reported in the initial cohorts, a recent review identified variability in the developmental phenotypes [14].

Here, we report the identification of a *de novo* noncoding *RARB* gene variant (c.157+1895G>A) associated with a complex microphthalmia phenotype and significant global developmental delay. Recent genome-wide association studies (GWAS) report that over 90% of disease-associated gene variants fall in noncoding regions, where they have the potential to disrupt canonical mRNA splicing or transcriptional regulation of gene expression [15, 16]. Through *in silico* analyses and *in vitro* functional studies, we propose a role for the variant in modulating *RARB* transcriptional regulation via alteration of a highly conserved intronic regulatory region, possibly resulting in *RARB* overexpression, elevated *FOXC1* expression, and significantly increased cell proliferation.

## 2. Methods

### 2.1. Variant Identification and In Silico Analysis for Pathogenicity.

Trio exome sequencing was performed through Psomagen (Rockville, MD) and analyzed using SVS and VarSeq software (Golden Helix, Bozeman, MT) as previously described, including analysis of known ocular genes, OMIM genes, and copy number variation analysis [17, 18]. General population data was obtained from gnomAD (v2.1.1 and v3.1.2) [19] with variant annotations including Combined Annotation Dependent Depletion (CADD- v1.4) [20] and Genomic Evolutionary Rate Profiling (GERP) scores [21, 22] obtained through VarSeq or UCSC Genome Browser (http://genome.ucsc.edu) [23, 24]. Trio analysis of variants with a read *depth* > 10, CADD *score* > 15, and *MAF* < 0.001 with <3 homozygotes (recessive) or fewer than 5 alleles (de novo, X-linked) in gnomAD v2 or v3 did not identify any strong candidates (Supplemental Table 1). Subsequently, trio genome sequencing was undertaken through Psomagen. Briefly, samples were prepared using the Illumina TruSeq Nano DNA library and sequenced using an Illumina HiSeq X sequencer. VCF and BAM files were generated using the Isaac Aligner and Isaac Variant Caller using human genome build hg19. Data was analyzed using VarSeq for rare variants fitting a *de novo*, X-linked, homozygous, or compound heterozygous inheritance pattern using the same criteria applied to exome data with the additional requirement of at least one coding variant for compound heterozygous. Variants of interest were manually reviewed, and low-quality or messy regions were excluded. The *RARB* variant was named based on reference sequence NM_000965.4. Pathogenicity was evaluated using several *in silico* prediction tools. Effect on splicing was assessed using Human Splicing Finder software (HSF; v3.1) [25], MaxEntScan [26], and SpliceAI (Gencode v36) [27]. Additional *in silico* analysis of the variant was completed in the UCSC Genome Browser (http://genome.ucsc.edu) [23, 24]. In order to maximize the additional tracks available for analysis, the coordinates of the variant were converted to hg38. Candidate *cis*-regulatory elements were identified through the ENCyclopedia of DNA Elements (ENCODE; [28] cCRE track) and confirmed through the H3K27Ac track; regions of chromatin interaction were identified with the Micro-C chromatin structure track [29]; regions associated with transcription factor DNA-binding profiles were identified with the JASPAR CORE collection (predicted) and ReMap (complied from over 7,500 publicly available ChIP-seq data sets) tracks; and sequence conservation was analyzed using PhyloP and Multiz Alignments of 100 Vertebrates tracks.

### 2.2. Cell Culture.

Human B3 lens epithelial cells (CRL-11421) and human embryonic kidney (HEK293) cells (CRL-1573) were obtained from ATCC (Manassas, VA). Human lens epithelial cells were maintained in minimal essential medium (MEM, Invitrogen, Waltham, MA) supplemented with 20% heat-inactivated fetal calf serum (FCS; HyClone, Logan, UT), 1X MEM nonessential amino acids (Gibco), 200 *μ*M L-glutamine (Gibco, Billings, MT), and 100 *μ*M sodium pyruvate (Gibco). HEK293 cells were cultured in Dulbecco’s Modified Eagle Medium (DMEM, Sigma, St. Louis, MO) supplemented with 10% heat-inactivated FCS (HyClone), 200 *μ*M L-glutamine (Gibco), and 100 *μ*M sodium pyruvate (Gibco).

### 2.3. Minigene Splicing Assays.

A 2.3 kb fragment that included exon 1 and 2,144 bp of intron 1–2, which contained the c.157+1895G>A variant, was PCR-amplified from the proband’s genomic DNA using Phusion high-fidelity polymerase (Thermo Fisher Scientific, Waltham, MA). NotI and XbaI restriction sites were appended at the 5′ and 3′ ends, respectively. All primers used are listed in Supplemental Table 2. Fragments were ligated into pcDNA3.1/V5-His TOPO TA expression vectors (Invitrogen) using the same restriction sites. Wild-type (WT) constructs and constructs harboring the intronic variant were identified via sequencing (Functional Biosciences, Madison, WI). Additionally, a second 584 bp fragment that included 435 bp of intron 1–2 and exon 2 was PCR-amplified from genomic DNA using Phusion high-fidelity polymerase (Thermo Fisher Scientific). XbaI and ApaI restriction sites were appended to the 5′ and 3′ ends, respectively (Supplemental Table 2). Fragments were inserted into pCR-II TOPO TA vectors (Invitrogen) and sequenced (Functional Biosciences). Using the appended restriction sites, the 584 bp fragment was excised from the pCR-II TOPO TA vector and ligated into the pcDNA3.1/V5-His TOPO TA expression constructs carrying either the WT or variant genomic sequence to generate the *RARB* minigene construct. Fully constructed WT and variant minigenes were validated via sequencing (Functional Biosciences).

Human lens epithelial or HEK293 cells grown to approximately 70% confluency in 6-well plates were transfected with 2 *μ*g of minigene DNA using Lipofectamine 2000 reagent (Invitrogen) according to the manufacturer’s protocol. Total RNA was harvested at 48 hours, and RNA was extracted using a Direct-Zol RNA Miniprep Kit (Zymo, Irvine, CA). For RT-PCR, cDNA was synthesized from 1 *μ*g total RNA using Oligo(dT) in a volume of 20 *μ*L and diluted 1 : 3 prior to use. A 2 *μ*L cDNA template was used in each 20 *μ*L reaction using the T7 primer to the pcDNA3.1 TOPO expression vector and to exon 2 (for minigene mRNA) or to exon 1 and exon 2 (for endogenous and minigene mRNA), and cellular glyceraldehyde-3-phosphate dehydrogenase (*GAPDH*, Supplemental Table 2). Bands were excised, purified using a Zymoclean Gel DNA Recovery Kit (Zymo), and sequenced (Functional Biosciences).

### 2.4. Regulatory Constructs and Luciferase Reporter Assay.

Luciferase reporter constructs were generated by subcloning synthesized *RARB* gBlock gene fragments (Integrated DNA Technologies, Coralville, IA) into a basic pGL4.10 [Luc2] vector (Promega, Madison, WI) using a ClonExpress II One Step Cloning Kit (Vazyme Biotech Co, San Diego, CA). Sequences for each of the gBlock gene fragments are shown in Supplemental Table 2. Specifically, 743 bp of the *RARB* promoter including the TSS (chr3:25427939–25428731 (hg19)) was inserted upstream of the firefly luciferase (Luc2) gene, and a 442 bp conserved region (CR1; chr3:25430445–25430886 (hg19)), with or without the *RARB* c.157+1895G>A variant, was inserted in a forward orientation directly downstream of the Luc2 gene. The 442 bp conserved region (CR1, chr3:25430445–25430886 (hg19)) includes the predicted 242 bp ENCODE element (ENCODE Accession: E2186460/enhD, chr3:25430545–25430786 (hg19)) harboring the *RARB* c.157+1895G>A variant (3:25472274-G-A (hg19)) surrounded both upstream and downstream by 100 bp of intronic sequence (Supplemental Figure 1). All constructs were verified by sequencing.

Human lens epithelial or HEK293 cells were plated in 24-well plates and transfected using Lipofectamine 2000 (Invitrogen) at a 1 : 2 DNA : lipid ratio, according to the manufacturer’s protocol. Each transfection included equimolar amounts of the respective pGL4 reporter plasmids (500 ng) and 10 ng of *Renilla* luciferase reporter plasmid (pGL4.74[hRluc/TK] Vector, Promega). Both Firefly and *Renilla* luciferase activities were quantitated within the same lysate for each sample after 48 hours using a dual luciferase reporter assay system (Promega). Firefly luciferase values were normalized to *Renilla* luciferase activity and expressed as a fold response compared with values calculated from activity driven by the *RARB* promoter only from three biological replicates, performed in triplicate.

### 2.5. RARB Overexpression.

To induce *RARB* overexpression, human lens epithelial cultures were transfected with either 500 ng human retinoic acid receptor beta (*RARB*) (NM_000965) in pCMV6-AC-GFP vector (*RARB* (tGFP-tagged); OriGene Technologies, Rockville, MD) or 500 ng control pcDNA3.1/V5-His TOPO TA expression vector (Invitrogen) using Lipofectamine 2000 (Invitrogen) as described above. Alternatively, recombinant human chemokine (C-C motif) ligand 28 (CCL28; PeproTech, Cranbury, NJ) was separately used to induce *RARB* overexpression, as it was previously shown to induce *RARB* gene expression in osteoclast precursors and oral squamous cell carcinoma (OSCC) cells [30]. CCL28 was prepared in sterile water and added directly to the culture media at a final concentration of 100 ng/mL. CCL28-supplemented media was exchanged after 24 hours. An equivalent volume of sterile water was added to control cultures. The treated cultures were divided with a portion of the culture used for quantification of cell growth and viability and another portion subjected to RT-qPCR for genes of interest.

### 2.6. Quantification of Cell Growth and Viability.

Total cell number, doubling rate, and viability were determined in *RARB* (tGFP-tagged) transfected cultures and CCL28-treated cultures 48 hours after treatment. Human lens epithelial cells were initially seeded at a density of 80,000 cells/well (22,857 cells/cm^2^) in 12-well plates; then, after 48 hours, cells were enzymatically detached, and final live-cell counts were determined using the Trypan Blue exclusion method. Doubling rate, the number of hours it takes for a population of cells to double their total cell number, was determined using an online doubling rate calculator (http://www.doubling-time.com/compute.php), using initial and final cell concentrations calculated by normalizing against surface area. Of note, there is an inverse relationship between doubling rate and cell proliferation, where a higher doubling rate indicates slower cell proliferation.

### 2.7. RT-qPCR.

Total RNA was extracted using Direct-zol RNA MiniPrep (Zymo) and treated with DNase I (Invitrogen) prior to cDNA synthesis. RNA concentration and purity were quantified using a NanoDrop ND2000 spectrophotometer (Thermo Fisher Scientific). cDNA was synthesized from 400 ng total RNA using Oligo(dT) priming with a SuperScript III Reverse Transcriptase Kit (Thermo Fisher Scientific). cDNA was used as a template in a 10 *μ*L reaction containing 10 *μ*L PowerTrack SYBR Green Master Mix (Applied Biosciences, Waltham, MA). Transcript-specific primers (Supplemental Table 2) were used to analyze select genes, using *B-ACTIN* (*ACTB*) as the reference gene for the quantification of relative expression levels via RT-qPCR. All RT-qPCR reactions were performed in duplicate on the CFX96 Touch Real-Time PCR Detection System (BioRad, Hercules, CA). A melt curve was generated for each primer set in order to verify the amplification of a single, specific product. Fold change in gene expression relative to expression in control cells from three independent replicates was calculated for each primer set using the 2^−*ΔΔCt*^ method [31].

### 2.8. Statistical Analysis.

All statistical analyses and graphical representations for the data presented were computed using GraphPad Prism 9 for Windows 64-bit, Version 9.3.1 (GraphPad Software; http://www.graphpad.com). Significant differences between groups were determined using either one-way analysis of variance (ANOVA) using Tukey’s post hoc multiple comparison test or Student’s unpaired *t*-test, as indicated in each figure. Data were derived from three independent replicates, each performed in duplicate (*n* = 6) or triplicate (*n* = 9), as indicated. *p* values < 0.05 were considered statistically significant.

## 3. Results

### 3.1. Variant Identification and In Silico Analysis for Pathogenicity.

**Figure 1:**
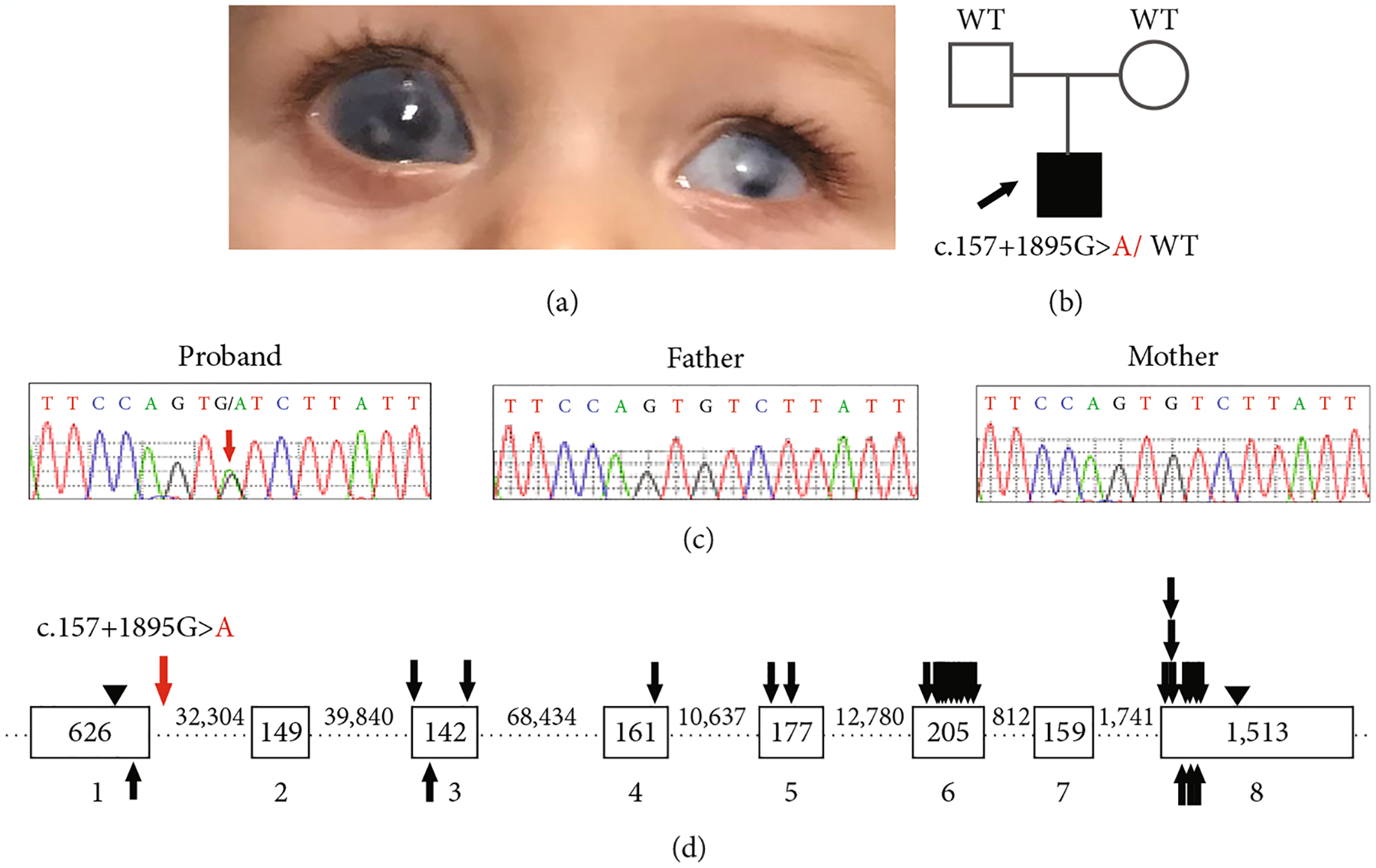
Identification of a de novo noncoding *RARB* gene variant (NM_000965.4: c.157+1895G>A). (a) Clinical image from the proband showing bilateral Peters anomaly and congenital glaucoma, right eye megalocornea and severe iris hypoplasia resembling aniridia, and left microphthalmia and microcornea. (b) Pedigree showing affected individual (arrow) and genetic testing results. (c) Sequence traces for proband and both parents (red arrow indicates the variant). (d) Schematic of the *RARB* gene including the location of the c.157+1895G>A variant within intron 1–2 (red arrow) and locations of previously reported human disease-associated variants (missense and truncation/frameshift coding variants are indicated with black arrows above and below the gene schematic, respectively). Exonic sequences are represented as open boxes; intronic sequences are represented as dashed lines; exon and intron sizes are indicated in base pairs. Start/stop codons are indicated with arrowheads. WT: wild-type allele.

A family consisting of unaffected parents and their 6-year-old affected son enrolled in the study. The affected individual displayed bilateral Peters anomaly (consisting of iris hypoplasia, corneal opacity, iridocorneal, and lenticular-corneal adhesions with lens opacity) and congenital glaucoma (Figure 1(a)). In his right eye, the iris hypoplasia was severe and resembled aniridia (with ciliary processes visible) along with megalocornea and posterior vitreous detachment; he ultimately lost all vision in the right eye due to injury. His left eye had microphthalmia and microcornea and has a visual acuity of light perception only. Nonocular anomalies included significant global delays, especially in language (nonverbal at 3 years old; 3-word sentences at 5–6 years of age), autism spectrum disorder, slow growth, umbilical hernia, retractile testes, and innocent heart murmur. Trio exome sequencing followed by trio genome sequencing did not identify any pathogenic alleles, including copy number variations (Supplemental Table 1). Genome sequencing identified one strong candidate variant: a *de novo* noncoding variant (3:25472274-G-A (hg19); NM_000965.4:c.157+1895G>A) in retinoic acid receptor beta (*RARB*) which was not present in 31,406 alleles in the gnomAD database (v2.1.1) but has since been reported in 1/152,134 alleles in gnomAD v3.1.2. Since this variant was also noted in one individual in the TOPMed population (rs1381205738) and gnomAD data includes TOPMed participants, the gnomAD variant likely represents the same allele. Given that TOPMed combines data from studies seeking to understand heart, lung, blood, and sleep (HLBS) disorders and the gnomAD database indicates that “individuals with severe disease may still be included in the data sets,” the individual in gnomAD may be affected. Unfortunately, clinical details are unavailable for this individual in gnomAD. The variant demonstrated high conservation for the affected nucleotide with a GERP score of 5, a PhyloP score of 2.34, and a CADD score of 18.75; the surrounding region also showed 79–98% conservation in multiple species over a 442 bp sequence, termed CR1 (conserved region 1; Supplemental Figure 1), supporting its potential functional role. Sanger sequencing confirmed the presence of the variant in the proband and its absence in both parents (Figures 1(b) and 1(c)). Additionally, the clinical features of the proband overlap previously reported phenotypes associated with dominant variants in *RARB* [13, 14, 32].

The MANE transcript of *RARB* (NM_000965.4) has eight exons and encodes a 448-amino acid protein. The identified G>A variant falls 1895 bp into the first intron (intron 1–2) of *RARB* (Figure 1(d)). Potential splicing disruption was evaluated via three *in silico* prediction tools: Human Splicing Finder (HSF; [25]), MaxEntScan [26], and SpliceAI [27]. Neither MaxEntScan nor SpliceAI predicted an effect for the c.157+1895G>A variant. However, HSF predicted activation of a cryptic donor site within intron 1–2 next to the site of the variant, strengthening recognition of the splice site by 26.39% (63.69 > 80) (Supplemental Table 3). Utilization of the aberrant splice site would likely extend Exon 1 by 1892 bp with the introduction of an erroneous stop codon, thus resulting in the translation of a truncated protein (82 amino acids) consisting of 52 amino acids derived from *RARB* exon 1 and 30 erroneous amino acids derived from intron 1–2. HSF also predicted that the presence of the c.157+1895G>A variant may alter auxiliary sequences responsible for regulating the splicing process, suggesting a second possible mechanism for a splicing effect.

**Figure 2:**
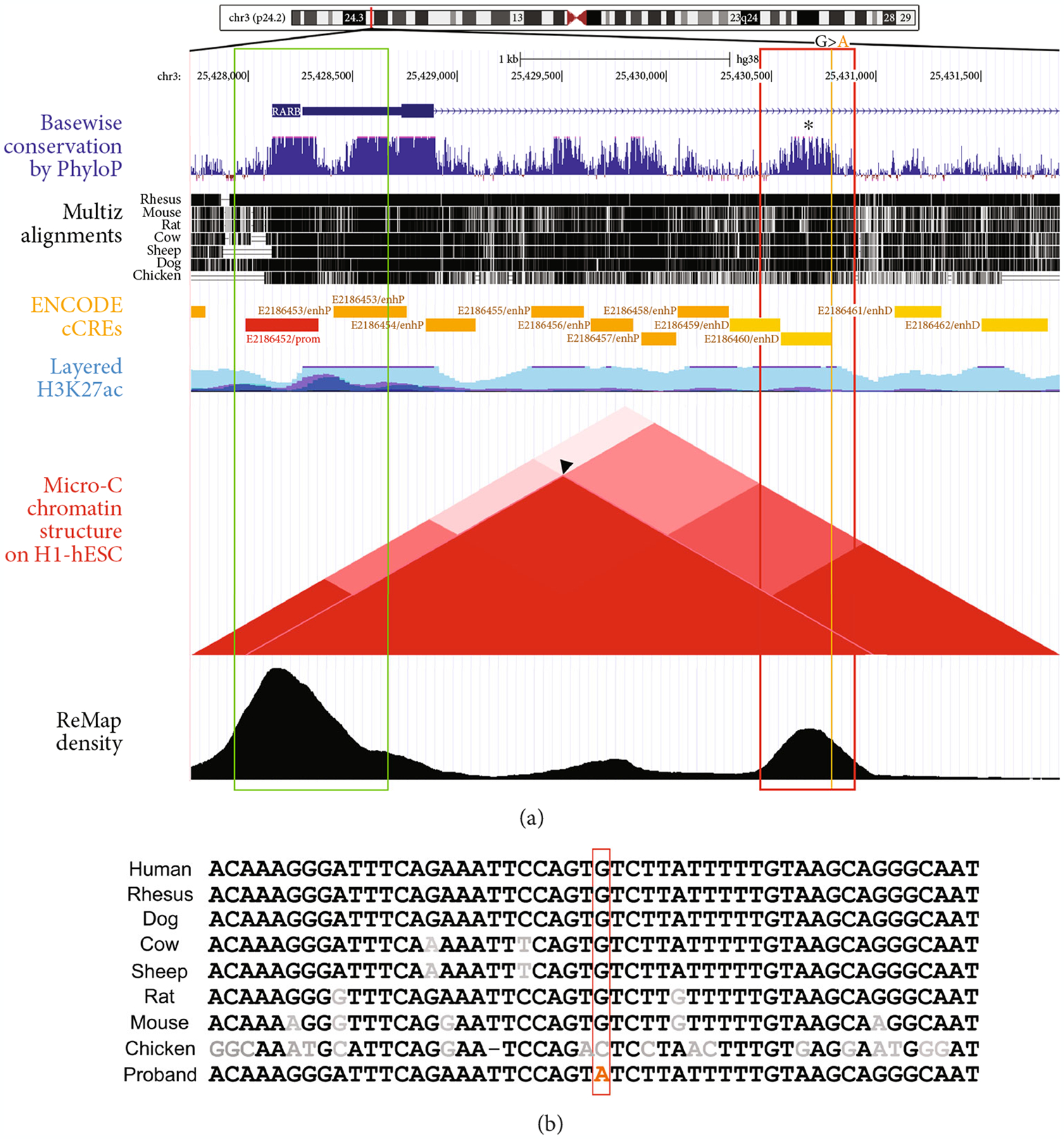
Assessment of pathogenicity by *in silico* analysis. (a) UCSC Genome Browser output highlighting a 4,143 bp region including the *RARB* gene promoter, exon 1, and the conserved region 1 (CR1) within *RARB* intron 1–2 carrying the c.157+1895G>A variant (hg38; variant coordinates 3:25430783). *RARB* promoter region included in the reporter construct is indicated with a green box; the CR1 region is shown as a red box with c.157+1895G>A variant marked with an orange line. PhyloP histogram and Multiz Alignment illustrate high conservation of CR1 (black asterisk). ENCODE track shows predicted promoter (red rectangle) and candidate *cis*-regulatory elements (orange rectangles) including E2186460/enhD element located within CR1 and overlapping the c.157+1895G>A variant. H3K27Ac track shows CR1 is acetylated. The triangle plot indicates areas of interaction identified within the region (adapted from the Micro-C chromatin track), including between CR1 and the *RARB* promoter region (arrowhead). ReMap track demonstrates enrichment of transcription factor binding sites within CR1. (b) A multispecies alignment of the 50 bp fragment of CR1 surrounding the c.157+1895G nucleotide (red box). Nucleotides conserved across all species are indicated in black, while divergent nucleotides are shown in grey.

As first introns frequently carry enhancer sequences crucial for gene regulation and chromatin organization [33–36], another possibility is that this variant may affect the regulation of *RARB* gene expression. Regions important in transcriptional regulation within the first introns show high conservation and increased histone modifications indicating active chromatin [33]. Consistent with this, the 442 bp CR1 region surrounding the variant showed strong conservation based on PhyloP histogram and multispecies alignment (Figures 2(a) and 2(b); Supplemental Figure 1). Examination of ENCODE tracks identified a candidate *cis*-regulatory element (cCRE) encompassing the *RARB* c.157+1895G>A variant within the larger CR1 region. ENCODE identifies and classifies putative regulatory elements in the human genome based on biochemical signatures supported by collective DNase hypersensitivity, histone modifications (H3K4me3 and H3K27ac), or CTCF-binding data. The *RARB* c.157+1895G>A variant is located within a 242 bp sequence denoted as having a distal enhancer-like signature (ENCODE Accession: E2186460/enhD; Figure 2(a); Supplemental Figure 1). Candidate *cis*-regulatory elements with this classification are located >2 kb from the annotated transcriptional start site (TSS) of a gene and display high DNase hypersensitivity, H3K4 trimethylation, and H3K27 acetylation signals, all associated with poised chromatin and/or transcriptional regulation. Under ENCODE, a “high signal” is defined as a maximum *Z*-score across all surveyed biosamples greater than 1.64, which corresponds to the 95^th^ percentile of a one-tailed test. The E2186460 element displayed maximum *Z*-scores of 3.82, 4.84, and 3.68 for DNase hypersensitivity, H3K4 trimethylation, and H3K27 acetylation, respectively. Interestingly, the DNase hypersensitivity for this region varied among tissue types with high DNase hypersensitivity identified in the developing fetal eye but not in HEK293 cells in the ENCODE database [28]. Notably, chromatin folding data from Micro-C XL, a variant of Hi-C, detected an interaction between the CR1/E2186460 and the *RARB* promoter region (arrowhead; Figure 2(a)) in human embryonic stem cells (hESC), and this region was also strongly associated with transcription factor DNA-binding profiles supported by functional analysis of transcriptional regulators complied from over 7,500 publicly available ChIP-seq data sets (ReMap) (Figure 2(a)), together highlighting a potential functional role for the element in *RARB* gene regulation specifically.

### 3.2. Analysis of the Effect of the RARB Intronic Variant on mRNA Splicing Using a Minigene Construct.

**Figure 3:**
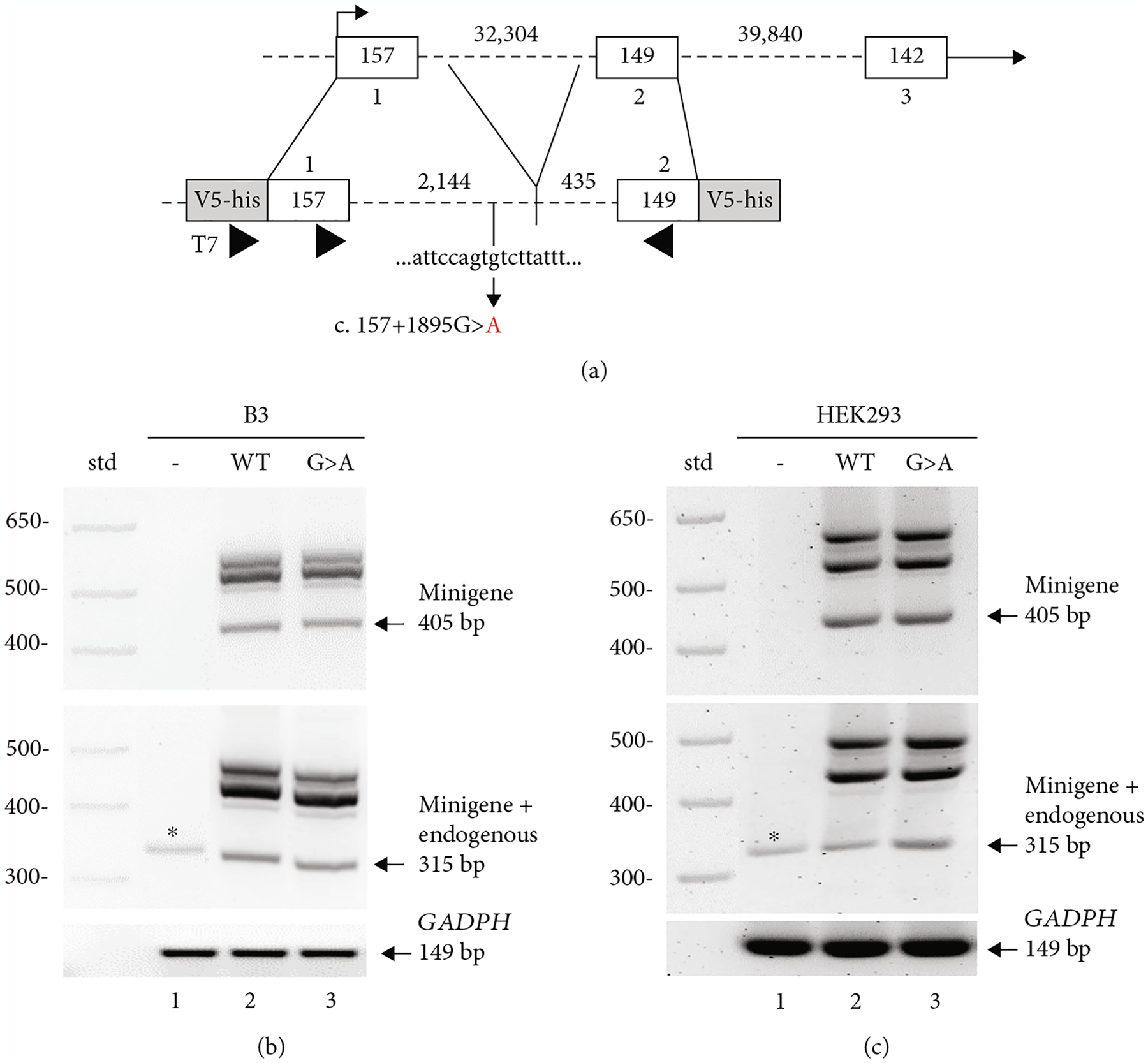
c.157+1895G>A variant has no apparent effect on the splicing of the *RARB* transcript. (a) Schematic of *RARB* minigene design. Exonic sequences are represented as open boxes; intronic sequences are represented as dashed lines; exon and intron sizes are indicated in base pairs. The *RARB* minigene contains the exon 1 coding sequence and 2,144 bp of the 5′ end of intron 1–2 ligated to 435 bp of the 3′ end of intron 1–2 and the exon 2 coding sequence. Positions of primers used for amplification of minigene-specific and endogenous RT-PCR products are shown (arrowheads). RT-PCR analysis of RNA isolated from transfected (b) human lens epithelial or (c) HEK293 cells showing endogenous and minigene-specific products. Cells were either mock transfected (-, lane 1) or transfected with wild-type (WT, lane 2) or variant (G>A, lane 3) minigene DNA. RNA was analyzed using primers that amplify minigene RNA only (top) or both minigene and endogenous *RARB* RNA (middle) or cellular *GAPDH* (bottom). Specific sizes of 1 kb standard marker (std) and expected band sizes for correctly spliced products are indicated.

Since one out of three *in silico* analyses (see above) predicted that the *RARB* c.157+1895G>A variant would activate a cryptic donor splice site in intron 1–2, potentially leading to an extension of exon 1, we performed the relevant functional studies. Patient samples were not available to directly analyze splicing since *RARB* is not expressed in the blood (GTex and Human Protein Atlas; [37, 38]). Given the length of intron 1–2 (32,304 bp), the potential impact of the c.157+1895G>A variant on the canonical splicing of the *RARB* mRNA transcript was evaluated using a minigene splicing assay in human cultured cells. Minigene constructs were designed against the coding region of exon 1 (157 bp) and exon 2 (149 bp) and contained an abridged intron 1–2 (Figure 3(a)). To retain the regulatory signals necessary for constitutive splicing at least 300 bp of the intron sequence surrounding the canonical 5′ and 3′ splice sites were included in the *RARB* minigene [39, 40].

Minigene splicing was analyzed in both human lens epithelial cells and human embryonic kidney (HEK293) cells. Cultured cells were transfected or mock transfected with 2 *μ*g of minigene DNA or pcDNA3.1 control plasmid, and RT-PCR was performed on RNA isolated after 48 hours. To ensure the detection of the minigene RNA, a primer complementary to the pcDNA3.1 vector sequence was used in combination with a primer specific to *RARB* exon 2. Correct splicing resulted in a 405 bp minigene-specific RT-PCR product observed in both cell types transfected with either WT or variant minigene constructs (Figures 3(b) and 3(c), top, lanes 2 and 3, arrow). As expected, no band was observed in control transfected cells using the minigene-specific primer set (Figures 3(b) and 3(c), top, lane 1). As *RARB* is expressed in both human lens epithelial cells and HEK293 cells, primers complementary to *RARB* exon 1 and exon 2 were used to detect both endogenous *RARB* and minigene expression resulting in a 315 bp RT-PCR product if correctly spliced. Correctly spliced endogenous *RARB* mRNA was detected in control transfected human lens epithelial cells and HEK293 cells (Figures 3(b) and 3(c), middle, lane 1, asterisk). A stronger band consistent with correct splicing of endogenous and minigene mRNA was observed in both cell types transfected with either WT or variant minigene constructs (Figures 3(b) and 3(c), middle, lanes 2 and 3, arrow). Finally, *GAPDH* expression demonstrated equivalent gel loading across samples (Figures 3(b) and 3(c), bottom).

We further observed a similar set of multiple larger products generated from both primer sets for both WT and variant minigene mRNA in human lens epithelial cells and HEK293 cells (Figures 3(b) and 3(c), top and middle, lanes 2 and 3). To detect potential differences in splicing between the WT and variant minigene mRNA, all products were excised and sequenced; however, no differences were identified between WT and variant sequences in either cell type. Overall, these results indicate that the c.157+1895G>A variant does not impact the splicing of the *RARB* mRNA transcript in the minigene in either cell type. While it is possible that the minigene design is masking the effect of the variant due to the loss of potentially relevant sequences which had to be removed given the considerable length of intron 1–2 (32,304 bp), the lack of an effect on splicing is consistent with the *in silico* predictions generated with SpliceAI, which has the highest accuracy of splice prediction tools [27, 41].

### 3.3. Assessing the Effect of c.157+1895G>A on the Transcriptional Regulation of RARB.

**Figure 4:**
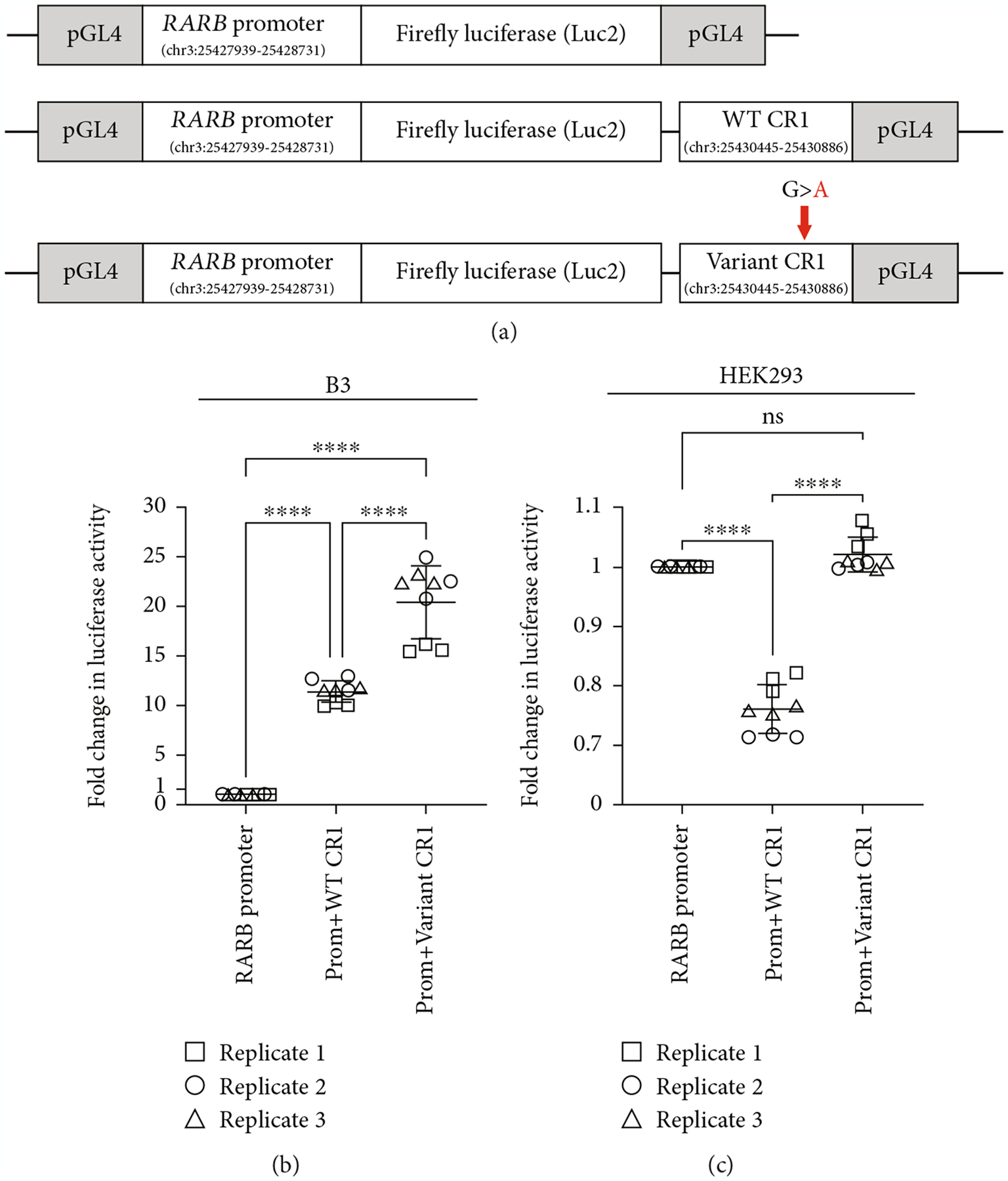
Assessing the effect of the c.157+1895G>A variant on the transcriptional regulation of *RARB*. (a) Schematic of luciferase (luc) reporter design. *RARB* promoter and the conserved region 1 (CR1), WT and variant (carrying c.157+1895G>A), were cloned into a basic pGL4.10 [Luc2] vector and transfected into human lens epithelial cells or HEK293 cells; chr3 coordinates for each *RARB* region are indicated based on hg19. Resultant fold change in luciferase activities from transfected (b) human lens epithelial or (c) HEK293 cells showed significantly increased activity in the presence of the CR1 harboring the c.157+1895G>A variant. Data were derived from three independent replicates, each performed in triplicate. One-way ANOVA with Tukey’s post hoc test was used to determine the statistical significance. ****p < 0.0001. ns = nonsignificant. Lines represent mean fold response. Error bars represent standard deviation.

As noted above, *in silico* analysis showed multiple lines of evidence supporting a role for the 442 bp region (CR1) encompassing the c.157+1895G>A variant in the regulation of *RARB*. To examine this possibility and how that might change in the presence of the c.157+1895G>A variant, we performed a dual luciferase reporter assay in human cultured cells. Several reporter constructs were generated, each consisting of the endogenous *RARB* promoter inserted into a pGL4 plasmid containing the firefly luciferase gene, alone or additionally carrying either the 442 bp WT CR1 region or one harboring the c.157+1895G>A variant (Figure 4(a); Supplemental Figure 1). Human lens epithelial cells or HEK293 cells were transfected with each of these constructs, along with a reporter plasmid carrying the *Renilla* luciferase gene, which served as a transfection control and in the normalization of luciferase activity.

Consistent with the strong conservation of CR1 and ENCODE predictions, we observed a significant increase in luciferase reporter activity in the presence of the putative WT regulatory element compared to the *RARB* promoter alone in lens epithelial cells (fold *change* = 11.40, *std.dev*. = 1.09, *p* value < 0.0001; Figure 4(b)). Moreover, reporter activity was further increased in the presence of the CR1 harboring the c.157+1895G>A variant when compared to either the *RARB* promoter alone (fold *change* = 20.39, *std.dev*. = 3.66, *p* value < 0.0001) or the WT CR1 (fold *change* = 1.78, *std.dev*. = 0.18, *p* value < 0.0001). Conversely, in HEK293 cells, luciferase reporter activity was markedly decreased in the presence of the WT CR1 compared to the *RARB* promoter alone (fold *change* = 0.76, *std.dev*. = 0.04, *p* value < 0.0001; Figure 4(c)). However, we observed an increase in luciferase reporter activity in the presence of the CR1 harboring the c.157+1895G>A variant when compared to the WT CR1 (fold *change* = 1.34, *std.dev*. = 0.05, *p* value < 0.0001), returning reporter activity to that observed with the promoter alone (fold *change* = 1.02, *std.dev*. = 0.03, *p* value = 0.2834). Together, these results support a role for the CR1 conserved sequence encompassing the variant position in modulating *RARB* promoter activity in two different cell types, acting as an enhancer in human lens epithelial cells but contributing to repression in HEK293 cells. In addition to suggesting a role in transcriptional regulation for the WT CR1 region, these results provide evidence indicating that the c.157+1895G>A variant may alter this region’s activity and lead to overexpression of *RARB*.

### 3.4. Induction of RARB Overexpression Results in Aberrant Cell Proliferation in Human Lens Epithelial Cells.

**Figure 5:**
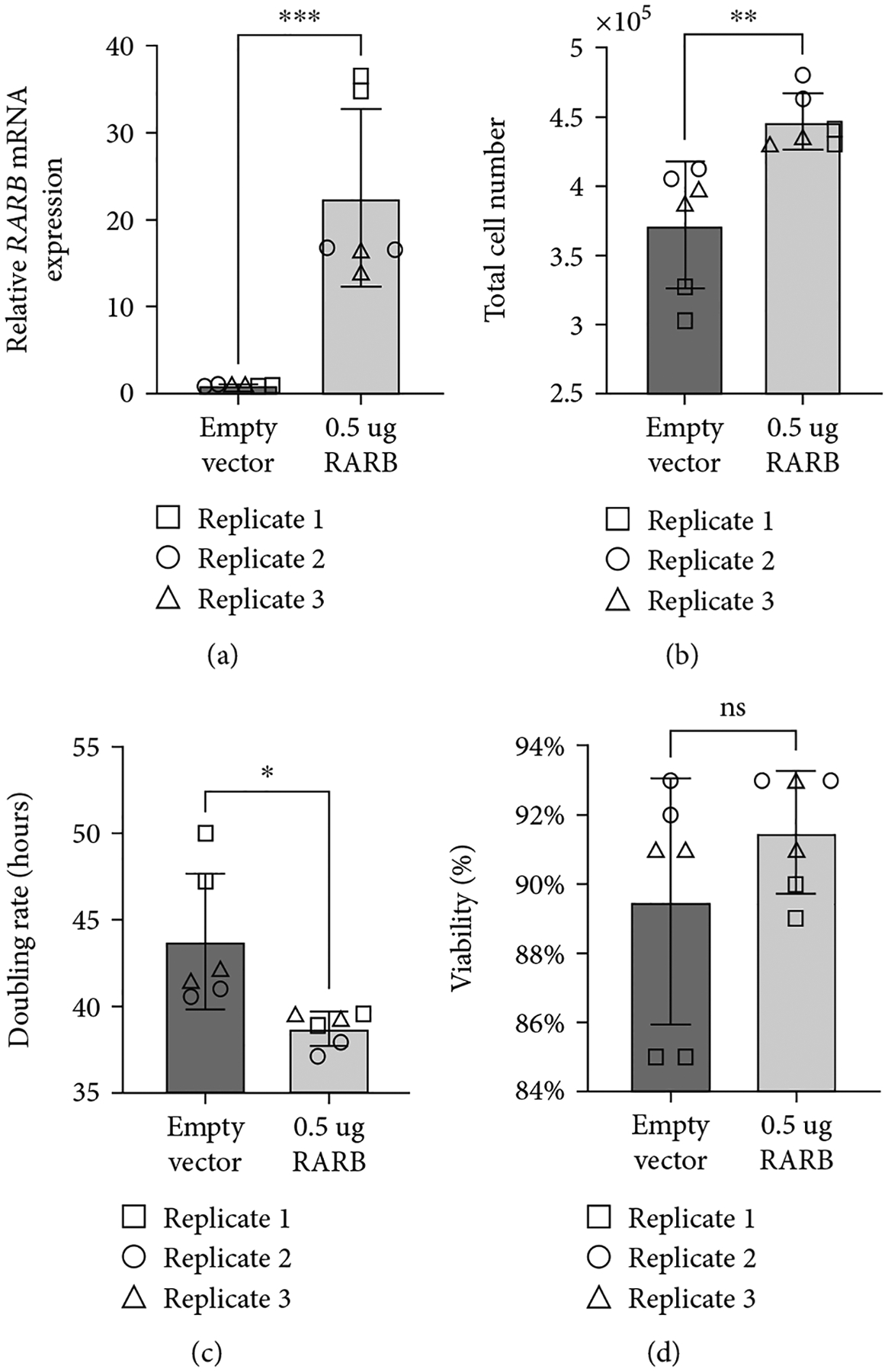
Overexpression of *RARB* leads to increased cell proliferation in human lens epithelial cells. (a) Relative changes in *RARB* mRNA expression were assessed via RT-qPCR. RNA was extracted from human lens epithelial cells transfected with either human *RARB* expression vector or empty pcDNA3.1/V5-His TOPO TA expression vector. (b) Total cell number, (c) doubling rate, and (d) viability were calculated using the Trypan blue exclusion method. Data were derived from three independent replicates, each performed in duplicate. Unpaired *t*-tests were used to determine the statistical significance of any differences between transfected and control transfected samples. *p < 0.05, **p < 0.01, and ***p < 0.001. ns = nonsignificant. Error bars represent standard deviation.

**Figure 6:**
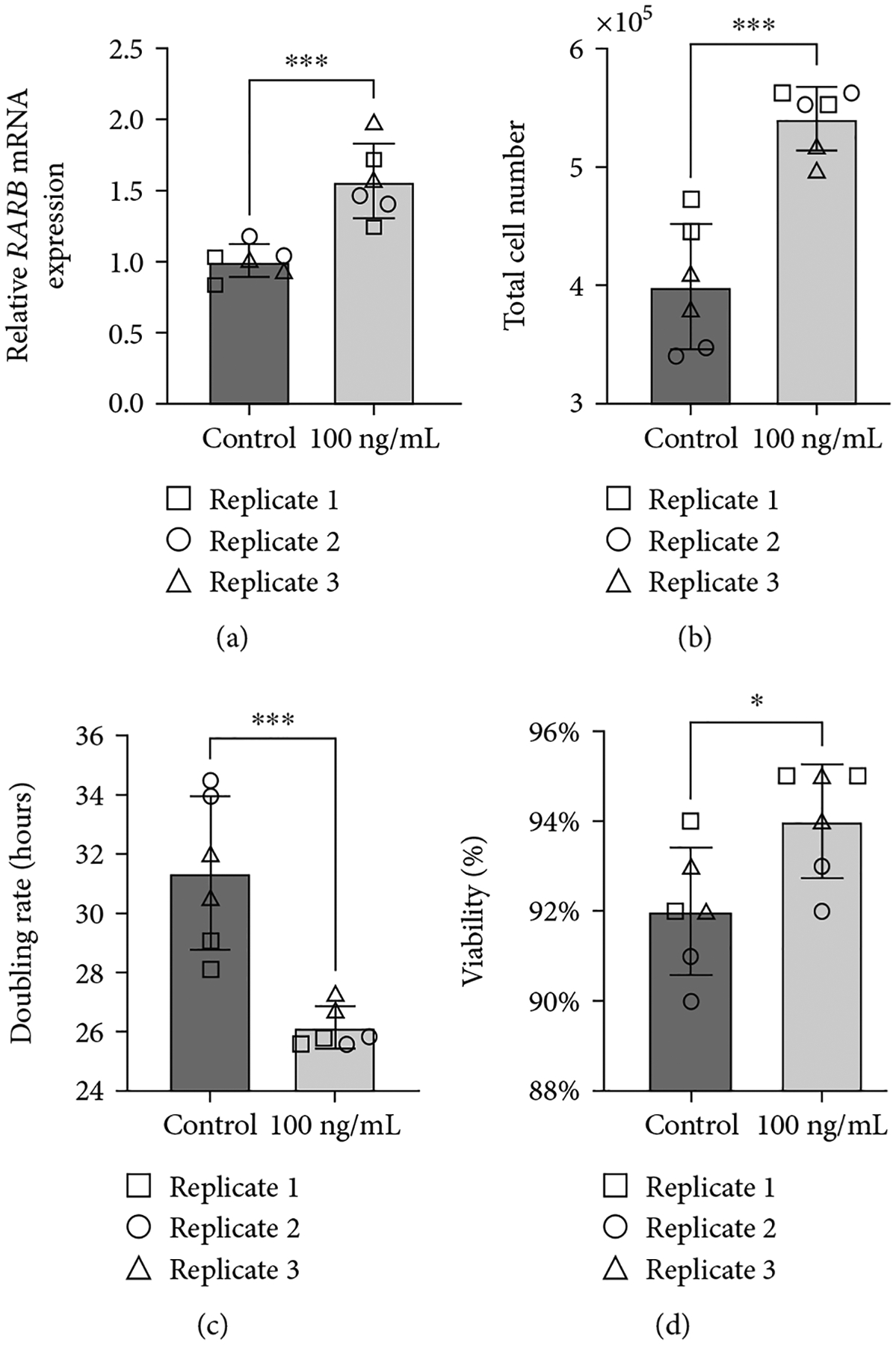
CCL28-induced *RARB* overexpression leads to increased cell proliferation and viability in human lens epithelial cells. (a) Relative changes in *RARB* mRNA expression were assessed via RT-qPCR. RNA was extracted from human lens epithelial cells treated with 100 ng/mL CCL28 or sterile water (control). (b) Total cell number, (c) doubling rate, and (d) viability were calculated using the Trypan blue exclusion method. Data were derived from three independent replicates, each performed in duplicate. Unpaired *t*-tests were used to determine the statistical significance of any differences between CCL28-treated and control samples. *p < 0.05 and ***p < 0.001. Error bars represent standard deviation.

To better understand the potential impact of the *RARB* c.157+1895G>A variant on ocular dysgenesis, we further explored the effect of *RARB* overexpression on cell growth, viability, and *RARB*-related gene expression in human lens epithelial cells. *RARB* overexpression was induced using two approaches: transfection of cells with a human *RARB* expression construct (Figure 5) or by treatment of cultured cells with chemokine (C-C motif) ligand 28 (CCL28) (Figure 6). In both cases, changes in total cell number, doubling rate, and viability were evaluated after 48 hours using a Trypan blue exclusion assay prior to RNA extraction. Relative changes in *RARB* mRNA expression as well as expression of downstream *RARB*-related gene targets, were interrogated via RT-qPCR.

As expected, we observed a significant increase in *RARB* mRNA expression in cells transfected with the *RARB* expression construct relative to control transfected cells (fold *change* = 22.52, *std.dev*. = 10.23, *p* value < 0.0004; Figure 5(a)). Overexpression of *RARB* resulted in increased total cell number (*p* value = 0.0044; Figure 5(b)) and a concurrent decrease in doubling rate (*p* value = 0.0123; Figure 5(c)) compared to control transfected cells. However, no change in cell viability was detected in transfected cultures overexpressing *RARB* (*p* value = 0.2460; Figure 5(d)). Together, these data indicate that *RARB* overexpression may promote aberrant cell proliferation during eye development, albeit without having an impact on overall cell health and survivability.

Consistent with previously published data, human lens epithelial cultures treated with 100 ng/mL CCL28 for 48 hours demonstrated a significant increase in *RARB* expression compared to untreated control cells (fold *change* = 1.564, *std.dev*. = 0.31, *p* value = 0.0007; Figure 6(a)). In addition, CCL28-induced *RARB* overexpression was positively correlated with a statistically significant increase in cell proliferation, as indicated by increased total cell number (*p* value = 0.0002; Figure 6(b)) and a decreased doubling rate (*p* value = 0.0008, Figure 6(c)). These results are similar to those observed in transfected cells overexpressing *RARB* (Figures 5(a)–5(c)) and further support a role for increased *RARB* expression in promoting aberrant cell proliferation during eye development. In contrast to transfected cultures, however (Figure 5(d)), overall cell viability was significantly increased in CCL28-treated cells compared to untreated controls (*p* value = 0.0273; Figure 6(d)). This difference in effect on cell viability between the *RARB* expression construct- and CCL28-mediated induction of *RARB* overexpression may be due to differences in the *RARB* expression level as *RARB* expression was 14 times higher in transfected cells compared to CCL28-treated cells. The difference may also be associated with CCL28 treatment acting on other pathways and not necessarily specific to *RARB* overexpression.

### 3.5. Overexpression of RARB Promotes Increased FOXC1 Expression in Human Lens Epithelial Cells.

To investigate the possible mechanisms by which *RARB* overexpression might disrupt ocular development, we evaluated the expression of established downstream targets of RA signaling known to play a role in vertebrate eye development. RA-related genes chosen for analysis included retinoic acid receptors *RARB*, *RARA*, and *RARG*, as well as the transcriptional regulators *PITX2* and *FOXC1* [42]. In addition, we investigated factors involved in Wnt/B-catenin signaling, including *WNT5A*/*5B*, *B-catenin*, *DKK2*, and *AXIN2*, and apoptosis (*EYA2*), all of which showed altered mRNA expression when RA signaling was disrupted in murine POM cells *in vivo* [10, 43, 44]. Relative changes in gene expression were interrogated via RT-qPCR from RNA extracted from the same human lens epithelial cultures either transfected with 0.5 *μ*g human *RARB* expression construct (Figure 5) or treated with 100 ng/mL CCL28 (Figure 6), allowing the above-described growth profiles to be correlated with any changes in gene expression.

**Figure 7:**
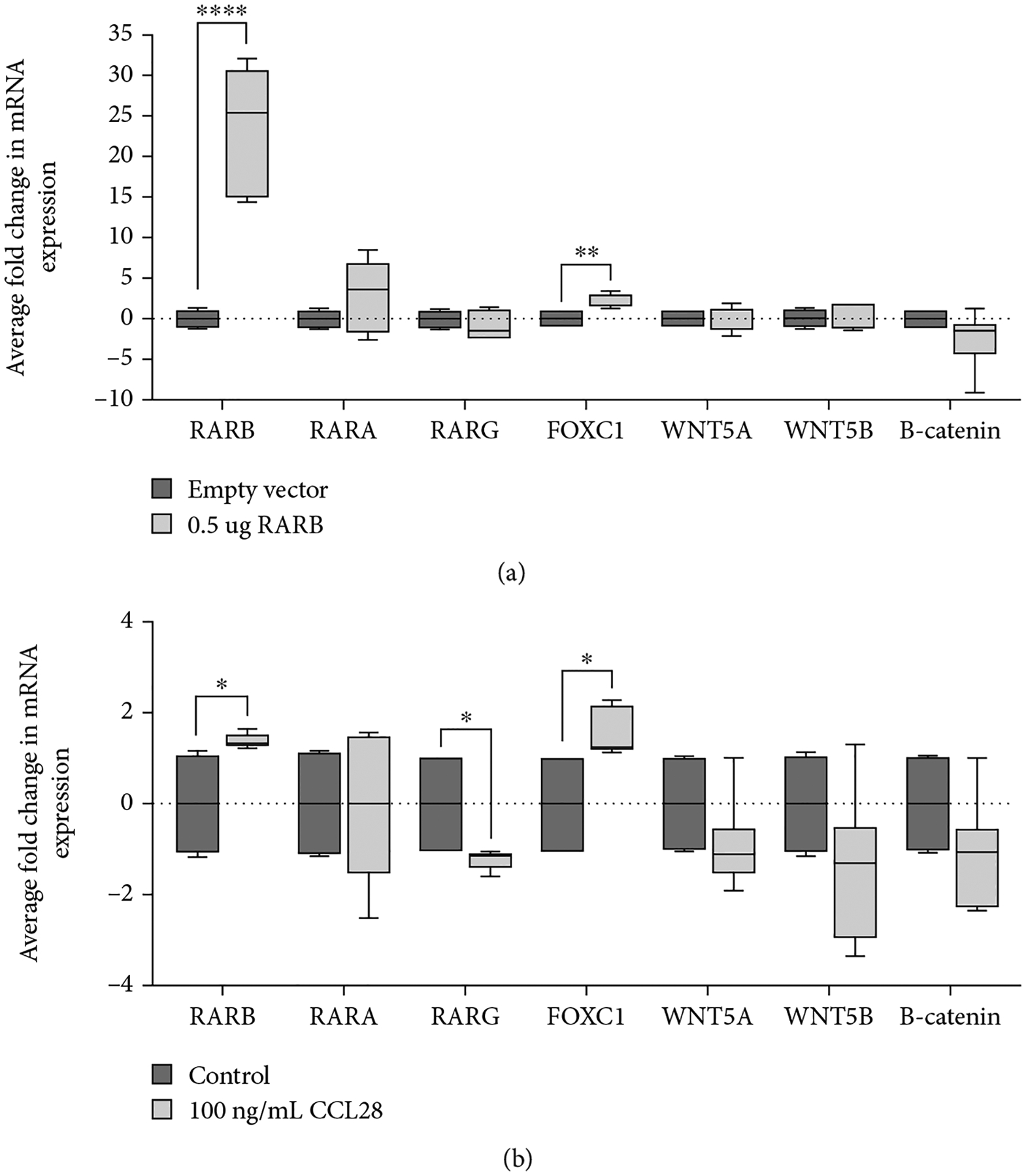
*RARB* overexpression leads to increased *FOXC1* expression. Relative changes in mRNA expression were evaluated in human lens epithelial cells (a) transfected with 0.5 *μ*g human *RARB* expression vector or (b) treated with 100 ng/mL CCL28. The expression level of several genes (*RARB*, *RARA*, *RARG*, *FOXC1*, *WNT5A*, *WNT5B*, and *B-catenin*) was measured via RT-qPCR. Values were normalized to *B-ACTIN* expression and presented as the average fold response compared to expression in control cultures. Data were derived from three independent replicates, each performed in duplicate. Unpaired *t*-tests were used to determine the statistical significance of any differences between transfected or CCL28-treated cells and their respective control samples. *p < 0.05, **p < 0.01, and ****p < 0.0001. Lines represent mean fold response. Error bars represent standard deviation.

As expected, *RARB* mRNA levels were significantly increased, confirming overexpression in both transfected cells (fold *change* = 23.79, *std.dev*. = 7.76, *p* value < 0.0001; Figure 7(a)) as well as those treated with CCL28 (fold *change* = 1.403, *std.dev*. = 0.15, *p* value < 0.017; Figure 7(b)). Moreover, *FOXC1* mRNA expression was significantly increased in both transfected cells (fold *change* = 2.06, *std.dev*. = 0.85, *p* value = 0.0049; Figure 7(a)) and CCL28-treated cells (fold *change* = 1.54, *std.dev*. = 0.52, *p* value < 0.014; Figure 7(b)) in comparison to expression in control cultures. However, *RARB* overexpression had no apparent effect on the expression of *RARA*, or *WNT5A*, *WNT5B*, and *B-catenin*, suggesting that the Wnt/B-catenin pathway may not play a significant role in the mechanism underlying *RARB* overexpression, at least in the context of human lens epithelial cells. Of note, *RARG* mRNA expression was significantly decreased uniquely in CCL28-treated cultures (fold *change* = −1.22, *std.dev*. = 0.22, *p* value < 0.027; Figure 7(b)), although it is not clear if the reduction in expression is directly due to *RARB* overexpression or an indirect effect of CCL28 treatment. The remaining genes (*PITX2*, *DKK2*, *AXIN2*, and *EYA2*) were below the level of detection in human lens epithelial cultures.

## 4. Discussion

In this study, we report the first noncoding variant in *RARB*, c.157+1895G>A, associated with a complex developmental ocular phenotype accompanied by significant global developmental delay, both consistent with previously reported features of MCOPS12 associated with *RARB* pathogenic variants. Given its location in the *RARB* gene, and our initial *in silico* analyses, we hypothesized that the variant might disrupt mRNA splicing or transcriptional regulation of *RARB*. While functional analysis of the variant did not identify a definitive splicing effect, our results suggest an effect for the variant on *RARB* transcriptional regulation (overexpression) through alteration of a conserved intronic regulatory region CR1. It is well-established that alterations in the expression and activity of *RAR*/*RXR* receptors, including *RARB*, lead to structural defects affecting a broad range of tissues, including the eye, heart, diaphragm, lungs, and limbs [1, 45]. Concerning eye development, transgenic mice expressing constitutively active *RARA* specifically in the lens exhibited microphthalmia, cataracts, and protrusion of aberrant lens tissue from the lens capsule [46], resembling the ocular defects observed with overexposure to retinoids *in utero* [47–49]. Induction of *RARB* overexpression in human lens epithelial cultures resulted in increased *FOXC1* mRNA expression and significantly increased cell proliferation. Gain-of-function *RARB* variants [13, 14, 32] and increased *FOXC1* gene dosage via gene duplication [50–53] have both been associated with developmental ocular phenotypes similar to those observed in the individual presented here, including microphthalmia, anterior segment anomalies, and congenital glaucoma. Therefore, it is possible that the identified *de novo* c.157+1895G>A variant alters the sensitive regulation of *RARB* levels, leading to aberrant transcriptional activity in response to retinoic acid and, as a result, the associated developmental abnormalities.

Notably, our results provide functional evidence supporting the regulatory activities of the CR1 region encompassing the candidate *cis*-regulatory element E2186460 predicted by ENCODE. The functional significance of *cis*-regulatory elements found within noncoding regions of developmental genes and their contribution to human ocular disorders has been previously described [54–56]. However, there is limited understanding of the *cis*-regulatory elements that control *RARB* expression and the specific factors that bind to these sequences that ultimately influence the development of a particular cell type. Interestingly, the effect of the WT CR1 differed between the two cell types tested, with reporter expression increased in lens cells but decreased in kidney cells. While cell type-specific differences in *cis*-regulatory element activity are not unexpected and are supported by varying DNase hypersensitivity for this element in differing cell types [28], further investigation into how this element plays a role in the regulation of *RARB* specifically in ocular cell types will be crucial to better understand *RARB* function in vertebrate eye development. Moreover, future biochemical assays may clarify which factors contribute to *RARB* expression and how the binding of those factors might be altered by the presence of the *RARB* intronic variant resulting in ocular and other developmental disorders.

Here, we report that induction of *RARB* overexpression utilizing two different approaches in lens epithelial cells resulted in significantly increased cell proliferation. Our results differ from that observed in carcinoma cell lines, where *RARB* has been identified as a tumor suppressor and induction of *RARB* expression, either through transfection [57–59] or treatment with pharmacological agents [30, 60, 61] promotes growth inhibition. However, it is plausible that *RARB* overexpression may exert differential effects in functionally mature or developmental cell types compared to cancerous cells, where differences in several factors, including chromatin accessibility, expression levels of corepressors and coactivators, and availability of cofactors, would ultimately influence the overall impact. In support of our results, aberrant upregulation of *RARB* expression in lingual epithelial cells during tongue development was associated with a hyperplastic lingual epithelium and overproduction of fungiform placodes containing oversized taste buds [62]. In addition, CCL28 was shown to directly stimulate the proliferation of cultured human hematopoietic stem cells (HSCs) and promote long-term maintenance of progenitor cell activity [63]. Although *RARB* levels were not directly assessed in this context, CCL28 was shown to induce *RARB* expression in both osteoclast precursors and OSCC cells [30]; therefore, it is likely to have a similar effect in human HSCs.

The augmented cell proliferation observed here is likely mediated by changes in downstream gene expression due to *RARB* overexpression. We observed a statistically significant increase in *FOXC1* expression in both *RARB*-transfected human lens epithelial cells and those treated with CCL28. Previous work indicates that precise levels of *Rarb* and *Foxc1* are required for proper eye development and that dysregulation of their expression can contribute to various ocular defects. Increased *FOXC1* expression due to gene duplication has been shown to result in anterior segment defects, including iris hypoplasia with glaucoma [50, 52], microcornea [51], and Peters anomaly [53], similar to the ocular features described here in the individual with a *de novo RARB* c.157+1895G>A variant. Notably, increased *FOXC1* expression has been shown to promote cell proliferation in several cancer cell types, including breast cancer [64], cervical cancer [65], and gastric cancer [66]. During ocular development, *FOXC1* represents an essential downstream gene target of RARB-mediated RA signaling in neural-crest-derived POM cells, which contribute to the formation of structures in the anterior segment of the eye, including portions of the lens, cornea, iris, and ciliary body [42]. Developmental ocular phenotypes particularly affecting the anterior segment are also seen in mice with haploinsufficiency or deletion of *Foxc1* [67, 68]. The temporal downregulation of *Foxc1* in murine POM cells was shown to be crucial for proper progression of corneal endothelium development, and knockdown or overexpression of *Foxc1* during this process similarly resulted in aberrant downregulation of genes associated with mesenchymal to epithelial transition (MET) initiation [69]. A reduction in *Foxc1* expression level was also reported in murine POM cells exhibiting a loss of *Rarb* and *Rarg*. Selective ablation of both RA receptors resulted in an abnormally thick layer of undifferentiated mesenchyme replacing the cornea and eyelids, hyperplasia of the primary vitreous body, as well as other anterior segment defects [10].

In summary, our study presents the identification of a de novo RARB c.157+1895G>A variant in an individual with a complex microphthalmia and provides functional data supporting the possible effect of this variant on *RARB* regulation (overexpression), which in turn likely promotes elevated *FOXC1* expression, and increased cell proliferation in *RARB*-expressing cells during development. Persistent *RARB* and *FOXC1* expression may disrupt the temporal expression patterns of downstream genes associated with the proper formation of structures in the developing eye. Moreover, alterations in expression may promote the maintenance of an enhanced proliferative state, potentially leading to the disruption of differentiation programs within RARB-expressing cells. These considerations, together with a characteristic phenotype observed in our patient and the lack of alternative explanation, support the possibility of the identified *de novo RARB* variant being involved in the patient’s phenotype. However, it should be noted that the same variant was also reported in 1/152,134 alleles in gnomAD v3.1.2, raising the possibility of variable expressivity/incomplete penetrance, or a coincidental finding. Further studies into the presented regulatory region, including screens in patient cohorts with features overlapping the *RARB* spectrum, will provide more decisive information about its possible involvement in human disease.

## Supplementary Material

Supplemental Materials_full Supplemental Table 1: rare variants identified by exome and genome sequencing.
Supplemental Table 2: list of primers and sequences used in this study.
Supplemental Table 3: impact predictions from Human Splicing Finder for RARB c.157+1895G>A.
Supplemental Figure 1: features of a 442 bp conserved region 1 (CR1). *(Supplementary Materials)*

## Data Availability

There are no other data associated with this manuscript. The *RARB* genetic variant has been submitted to ClinVar (SCV004231891).
