# Supplementary material for: A De Novo Noncoding RARB Variant Associated with Complex Microphthalmia Alters a Putative Regulatory Element
Source: Hum Mutat. 2024 Jan 27;2024:6619280. doi: 10.1155/2024/6619280 (PMC11501074; doi:10.1155/2024/6619280)

SUPPLEMENTARY MATERIALS

Supplemental Table 1. Rare variants identified by exome and genome sequencing.

Position	Gene	Variant	WES Reads	WGS Reads	Inheritance	CADD score	REVEL score	gnomAD v3	OMIM phenotype
Variants identified by trio exome sequencing									
12:118582499	PEBP1 (NM_002567.4)	c.455C>T p.(Ala152Val)	13/33	15/26	*De novo*	22.3	0.094	2/152,114	None
1:44057557	PTPRF (NM_002840.5)	c.1606C>G p.(Pro536Ala)	45/85	14/29	CH: Maternal	19.47	0.117	68/152,160	? Athelia (AR PTC)
1:44058233	PTPRF (NM_002840.5)	c.1774G>A p.(Val592Ile)	64/112	10/20	CH: Paternal	24.2	0.102	134/152,038	? Athelia (AR PTC)
1:44087628	PTPRF (NM_002840.5)	c.5678G>A p.(Arg1893His)	75/155	16/32	CH: Paternal	27.4	0.613	101/152,166	? Athelia (AR PTC)
X:72673444	CDX4 (NM_005193.2)	c.596_598delCCA p.(Thr199del)	18/18	20/20	XL: Maternal	18.41	NA	1/111,611	None
X:138286342	FGF13 (NM_001139500.2)	c.-73A>T	64/64	17/17	XL: Maternal	21.3	NA	7/111,060	Seizures and global delay (XLR MIS)
Additional variants identified by trio genome sequencing									
**3:25472274**	**RARB (NM_000965.4)**	**c.157+1895G>A**	-	**14/29**	***De novo***	**18.75**	**NA**	**1/152,134**	**Syndromic microphthalmia (AD MIS; AR PTC)**
11:100830655	ARHGAP42 (NM_152432.4)	c.1204A>G p.(Ile402Val)	-	9/13	CH: Maternal	25.4	0.279	1/152,154	None
11:100763695	ARHGAP42 (NM_152432.4)	c.385-20488A>G	-	22/43	CH: Paternal	17.75	NA	NP	None
2:160042312	TANC1 (NM_033394.3)	c.2521G>A p.(Ala841Thr)	-	12/33	CH: Maternal	22.4	0.307	15/152,152	None
2:159955772	TANC1 (NM_033394.3)	c.259+1426A>G	-	7/18	CH: Paternal	21.4	NA	117/152,156	None
X:129507168	SLC25A14 (NM_001282195.2)	c.*244A>T	-	14/14	Maternal	15.56	NA	1/112,015	None

Filtered for read depth >10, CADD (v1.4) score >15, at least one coding variant for compound heterozygous, and frequency in gnomAD v2 or v3 of <0.001 MAF and <3 homozygotes for recessive or <5 alleles for de novo and X-linked; Intergenic variants and low quality/messy regions excluded. AD, autosomal dominant; AR, autosomal recessive; MIS, missense; PTC, premature truncation.

Supplemental Table 2. List of primers and sequences used in this study.

**Sequencing**	**Forward**	**Reverse**	**Product**
h*RARB* Seq_Fwd6/Rev5	GGAGAGCGAGCGGTGAATGT	AAGTTGGGGTGTGGGATTCA	227
**Minigene design**	**Forward**	**Reverse**	**Product**
h*RARB* Exon1_Intron1	CATGGCGGCCGCAAGGGAGATCATGTTTGACTGTAT	CATGTCTAGATGAACCAAACACTGCATGGC	2301
h*RARB* Intron1_Exon2	CATGTCTAGAGGTTAAGTGAGCAACAAGGTCA	ATATGGGCCCCTTACATCCCTCACAGGCG	584
T7	TAATACGACTCACTATAGGG		
RARB	AAGGGAGATCATGTTTGACTGTAT	CTTACATCCCTCACAGGCG	316
GAPDH	CCAAGGTCATCCATGACAACT	GAGGCAGGGATGATGTTCTG	148
**RT-qPCR Target Gene**	**Forward**	**Reverse**	**Product**
RARB	AAGGGAGATCATGTTTGACTGTAT	CTTACATCCCTCACAGGCG	316
PITX2	ACATGTCCACACGCGAAGAA	GGTACATGTCGTCGTAGGGC	183
FOXC1	TTCGAGTCACAGAGGATCGG	CGGCTTTGAGGGTGTGTCAA	148
RARA	GGGGGAATCCTGAATCGAGC	GATGTCTCAAGAGCCGGTCC	234
RARG	CAGGTTTCCCCTTCGCCTTC	ATGGCTTGTAGACCCGAGGA	201
EYA2	CAACGTCCCCAACCAGAGTT	ACGGGTCACTGCTCCTCTTA	152
DKK2	CATCAAGTCCTCTCTGGGCG	GGACTGTGGCAATACCTCCC	168
WNT5A	AGTCGCCCCGGAACTTATTG	CTCCGAGCTCTCGCCCTTC	92
WNT5B	ATGGCCTACATAGGGGAGGG	GTGAAGGCGGTCTCTCGG	146
CTNNB1	TACTGGCTAGTGGTGGACCC	AGGTGAAGTCCTAAAGCTTGC	166
AXIN2	TAACCCCTCAGAGCGATGGA	AGTTCCTCTCAGCAATCGGC	143
B-ACTIN	GGACTTCGAGCAAGAGATGG	AGCACTGTGTTGGCGTACAG	234

***RARB* sequences included in reporter constructs (variant position is shown in red)**		**Product**
RARB promoter	GGAGCAGCGTCCCGGCTCCTCCCCTGCTCATTTTAAAAGCACTTCTTGTATTGTTTTTAAGGTGAGAAATAGGAAAGAAAACG<br>CCGGCTTGTGCGCTCGCTGCCTGCCTCTCTGGCTGTCTGCTTTTGCAGGGCTGCTGGGAGTTTTTAAGCTCTGTGAGAATCCT<br>GGGAGTTGGTGATGTCAGACTAGTTGGGTCATTTGAAGGTTAGCAGCCCGGGTAGGGTTCACCGAAAGTTCACTCGCATAT<br>ATTAGGCAATTCAATCTTTCATTCTGTGTGACAGAAGTAGTAGGAAGTGAGCTGTTCAGAGGCAGGAGGGTCTATTCTTTGC<br>CAAAGGGGGGACCAGAATTCCCCCATGCGAGCTGTTTGAGGACTGGGATGCCGAGAACGCGAGCGATCCGAGCAGGGTTT<br>GTCTGGGCACCGTCGGGGTAGGATCCGGAACGCATTCGGAAGGCTTTTTGCAAGCATTTACTTGGAAGGAGAACTTGGGAT<br>CTTTCTGGGAACCCCCCGCCCCGGCTGGATTGGCCGAGCAAGCCTGGAAAATGGTAAATGATCATTTGGATCAATTACAGGC<br>TTTTAGCTGGCTTGTCTGTCATAATTCATGATTCGGGGCTGGGAAAAAGACCAACAGCCTACGTGCCAAAAAAGGGGCAGA<br>GTTTGATGGAGTTGGGTGGACTTTTCTATGCCATTTGCCTCCACACCTAGAGGATAAGCACTTTTGCAGACATTCAGTGCAAG<br>GGAGATC	743
Putative WT element	CATGTTTTTTAACAGAAGGAAAAAATATGCTCAGGATCCTCGCCAAAAGTTATTAGACCTAATAGAGTTTTTACATGTAGACA<br>TTCTTCCTTTTGTAATTAACCTTCCAAAGATGCCTATTAAGTTGTAAGAGTAGGGGGAAAAGCTTTGTTTGTTCTTGGACAAGT<br>TAAACAAGGGATTTGCTTTATCTAGGGAGAGCGAGCGGTGAATGTGTCATTCGCCTAAAACTACCCGATAAGCAAGAAGTGT<br>TGATAACAGCAAAGTTTAGCTAATCAAAGAAACTTGCATTAGACAAAGTAAATAAAGGCATTTGACAAAGGGATTTCAGAAA<br>TTCCAGT**G**TCTTATTTTTGTAAGCAGGGCAATTAGTTCTTATTGTGGTCAGTTTCACAAGAGTTTGAATCCCACACCCCAACTT<br>TAGGTTAAATGAATAAAATGTAAATAT	442
Putative variant element	CATGTTTTTTAACAGAAGGAAAAAATATGCTCAGGATCCTCGCCAAAAGTTATTAGACCTAATAGAGTTTTTACATGTAGACA<br>TTCTTCCTTTTGTAATTAACCTTCCAAAGATGCCTATTAAGTTGTAAGAGTAGGGGGAAAAGCTTTGTTTGTTCTTGGACAAGT<br>TAAACAAGGGATTTGCTTTATCTAGGGAGAGCGAGCGGTGAATGTGTCATTCGCCTAAAACTACCCGATAAGCAAGAAGTGT<br>TGATAACAGCAAAGTTTAGCTAATCAAAGAAACTTGCATTAGACAAAGTAAATAAAGGCATTTGACAAAGGGATTTCAGAAA<br>TTCCAGT**A**TCTTATTTTTGTAAGCAGGGCAATTAGTTCTTATTGTGGTCAGTTTCACAAGAGTTTGAATCCCACACCCCAACTT<br>TAGGTTAAATGAATAAAATGTAAATAT	442

Supplemental Table 3. Impact predictions from Human Splicing Finder for *RARB* c.157+1895G>A.

Type	Interpretation		
New Donor splice site	Activation of a cryptic Donor site.<br>Potential alteration of splicing (cryptic exon activation)		
**Algorithm/Matrix**	**Position**	**Sequences**	**Variation**
HSF Donor site (matrix GT)	chr3:25430778	-REF: CCAGTGTCT<br>-ALT: CCAGTaTCT	63.69 > 80.5 (26.39%)

Type	Interpretation	
Alteration of auxiliary sequences	Significant alteration of ESE / ESS motifs ratio (2)	
**Algorithm/Matrix**	**Position**	**Sequence**
EIE (ESE Site Broken)	chr3:25430778	CCAGTG
Sironi_motif1 (ESS Site Broken)	chr3:25430778	CCAGTGTC
ESS_hnRNPA1 (ESS Site Broken)	chr3:25430779	CAGTGT
IIE (ESS Site Broken)	chr3:25430779	CAGTGT
ESE_SRp55 (ESE Site Broken)	chr3:25430780	AGTGTC
IIE (ESS Site Broken)	chr3:25430781	GTGTCT
PESS (New ESS Site)	chr3:25430782	TaTCTTAT
EIE (New ESE Site)	chr3:25430783	aTCTTA

Supplemental Figure 1. Features of a 442-bp Conserved Region 1 (CR1). Multiz alignment demonstrates high conservation of the entire CR1 sequence across select species; dark blue indicates highly conserved and light blue less conserved nucleotides. ENCODE candidate *cis*-regulatory elements (orange rectangles) and JASPAR regions enriched in transcription factor DNA-binding sites (TFBS) (grey rectangles) are indicated based on UCSC Genome Browser. CR1 encompasses the full candidate *cis*-regulatory element E2186460/enhD (242 bp; dark orange rectangle) surrounded by 100 bp of upstream and downstream sequences (blue rectangles). Please note that *RARB* c.157+1895G>A (red box/ font) shows high conservation and falls within both the predicted *cis*-regulatory element E2186460/enhD and JASPAR TFBS- enriched region.

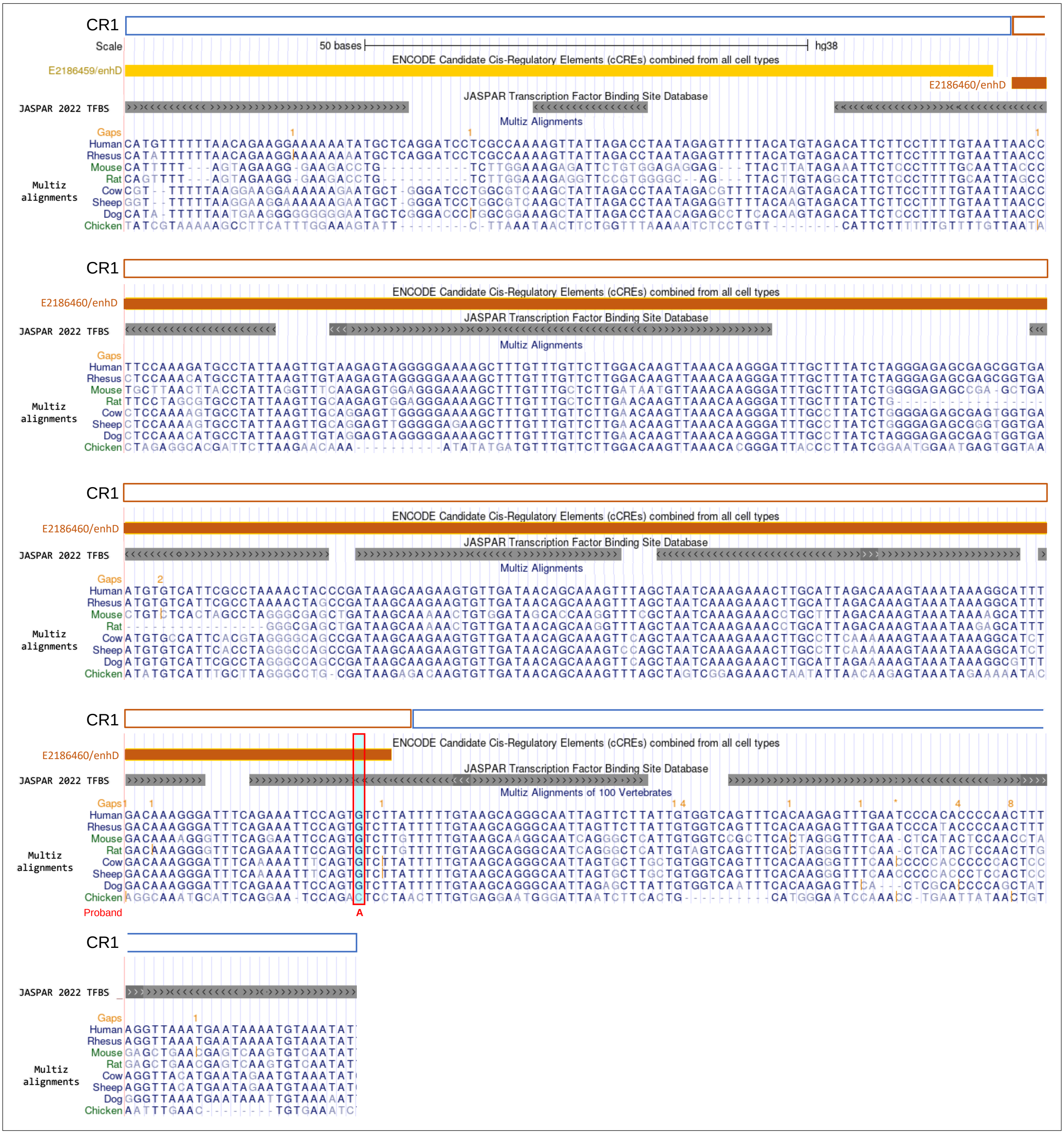

Supplement: Supplementary Materials — Supplemental Table 1: rare variants identified by exome and genome sequencing. Supplemental Table 2: list of primers and sequences used in this study. Supplemental Table 3: impact predictions from Human Splicing Finder for RARB c.157+1895G>A. Supplemental Figure 1: features of a 442 bp conserved region 1 (CR1). [file 6619280.f1.pdf]
